# Prognostic value of creatinine-to-cystatin c ratio in patients with type 2 diabetes mellitus: a cohort study

**DOI:** 10.1186/s13098-022-00958-y

**Published:** 2022-11-23

**Authors:** Wen Wei, Shanggang Li, Jin Liu, Yong Liu, Kaihong Chen, Shiqun Chen, Mei Tu, Hong Chen

**Affiliations:** 1 Department of Endocrinology, Zhujiang Hospital, Southern Medical University, Guangzhou, China; 2 Department of Endocrinology, Fujian Longyan First Hospital, Longyan First Affiliated Hospital of Fujian Medical University, Longyan, China; 3 Department of Cardiology, Guangdong Cardiovascular Institute, Guangdong Provincial People’s Hospital, Guangdong Academy of Medical Sciences, Guangzhou, China; 4 Department of Guangdong Provincial Key Laboratory of Coronary Heart Disease Prevention, Guangdong Cardiovascular Institute, Guangdong Provincial People’s Hospital, Guangdong Academy of Medical Sciences, Guangzhou, China; 5 Department of Cardiology, Guangdong Provincial People’s Hospital, School of Medicine, South China University of Technology, Guangzhou, China; 6 Department of Cardiology, Guangdong Provincial People’s Hospital, Affiliated South China Hospital, Southern Medical University, Guangzhou, China; 7 Department of Cardiology, Fujian Longyan First Hospital, Longyan First Affiliated Hospital of Fujian Medical University, Longyan, China; 8 Global Health Research Center, Guangdong Provincial People’s Hospital, Guangdong Academy of Medical Science, Guangzhou, China

**Keywords:** Low muscle mass, Creatinine-to-cystatin C ratio, Type 2 diabetes mellitus, Prognostic

## Abstract

**Background:**

The serum creatinine-to-cystatin C ratio (Scr/Scys) has been suggested as a surrogate marker of muscle mass and a predictor of adverse outcomes in many diseases. However, the prognostic value of Scr/Scys in patients with type 2 diabetes mellitus (T2DM) is unknown. The aim of this study is to assess the prognostic value of Scr/Scys in patients with T2DM.

**Methods:**

In this retrospective observational study, we enrolled 3668 T2DM patients undergoing coronary angiography (CAG). Serum creatinine (Scr) and serum cystatin C (Scys) levels were measured at admission. The study population was separated into low muscle mass (low-MM) and normal muscle mass (normal-MM) groups by Scr/Scys cut-off point. The association between muscle mass and long-term all-cause mortality was examined using Cox regression analysis.

**Results:**

During a median follow-up of 4.9 (3.0–7.1) years, a total of 352 (9.6%) patients died. The mortality was higher in patients with low-MM as compared with patients with normal-MM (11.1% vs. 7.3%; p < 0.001). Low muscle mass was associated with increased risk for long-term all-cause mortality, regardless of whether Scr/Scys were used as a continuous variable (adjusted hazard ratio: 1.08 [95% confidence interval (CI) 1.03 to 1.13]; p = 0.009) or a categorial variable (adjusted hazard ratio: 1.36 [95% CI 1.03 to 1.75]; p = 0.021).

**Conclusion:**

Low muscle mass assessed by Scr/Scys was associated with increased risk of long-term all-cause mortality in diabetic patients.

**Supplementary Information:**

The online version contains supplementary material available at 10.1186/s13098-022-00958-y.

## Background

Sarcopenia is age-related degenerative skeletal disease characterized by a progressive and generalized reduction in muscular mass, strength, and function [1]. The prevalence of sarcopenia is significantly higher in diabetics than in non-diabetics [2]. In diabetics, sarcopenia is associated with poor outcomes, such as albuminuria, chronic kidney disease (CKD), diabetic retinopathy (DR), diabetic peripheral neuropathy (DPN), atherosclerosis, cognitive impairment, infection and mortality [3–9].

The presence of low muscle mass (low-MM) is the core component of the algorithm to diagnose sarcopenia. Magnetic resonance imaging (MRI), computed tomography (CT) and dual energy X-ray absorptiometry (DEXA) are used to measure the quantity of muscle mass [1]. However, these methods require specific devices and have limitations such as exposure to radiation and lack of cost-effectiveness [10]. Bioelectrical impedance analysis (BIA) is a cost-effective measurement of muscle mass but is adversely influenced by the volume status of patients [11]. Recently, the serum creatinine-to-cystatin C ratio (Scr/Scys), also known as the sarcopenia index (SI), is suggested as a surrogate marker of muscle mass in patients with type 2 diabetes mellitus (T2DM), chronic obstructive pulmonary disease (COPD) and cancer [12–14]. Furthermore, Scr/Scys is also a predictor of adverse outcomes in patients with acute kidney injury (AKI), coronary artery disease (CAD), acute ischemic stroke, COPD and non-alcoholic fatty liver disease (NAFLD) [13, 15–18]. However, the relationship between Scr/Scys and clinical outcome in diabetic patients is unclear.

Therefore, we aim to evaluate the prognostic value of Scr/Scys for long-term mortality risk in patients with T2DM.

## Methods

### Study population

**Fig. 1:**
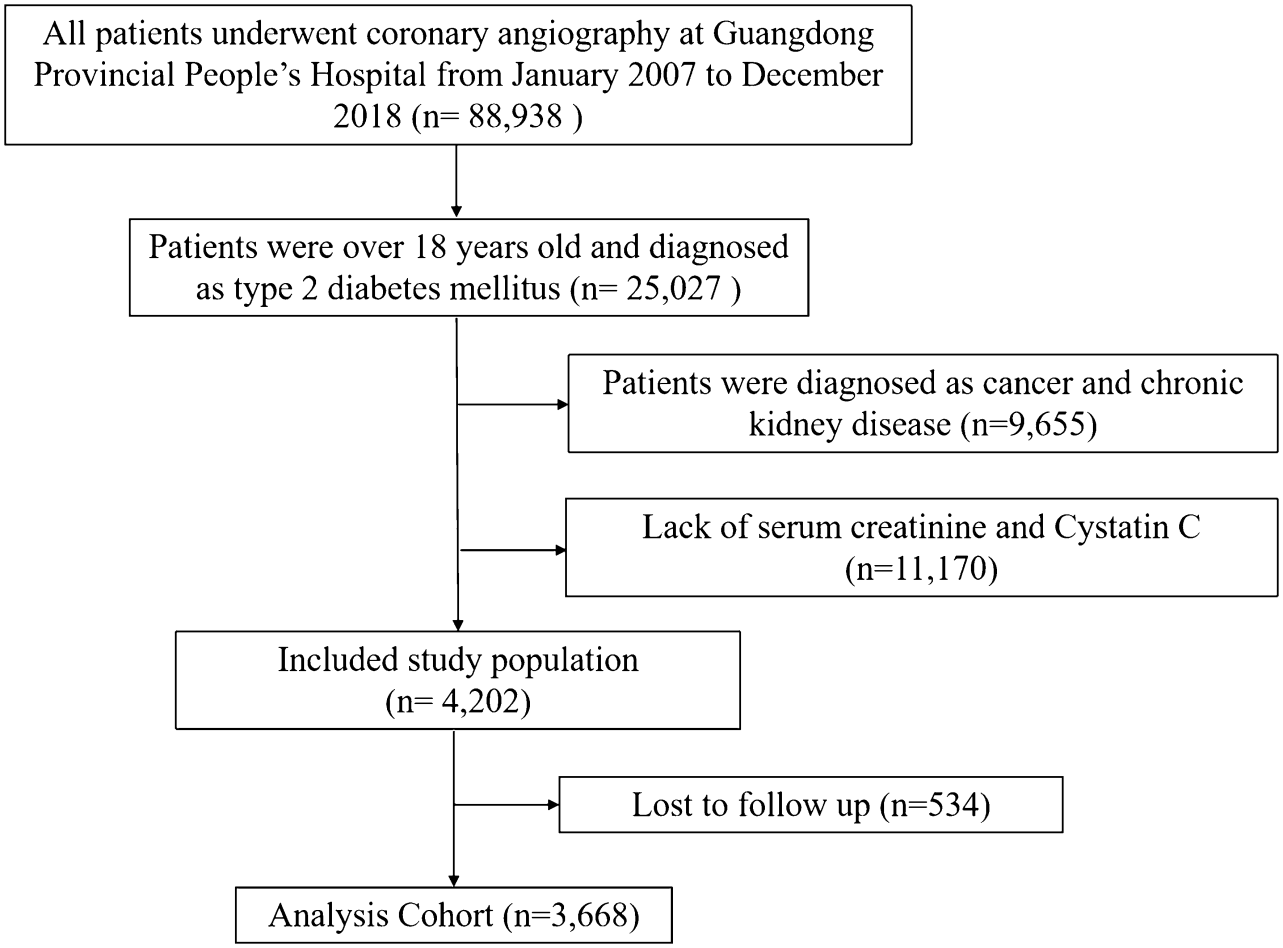
The flow of participants through the trial

The present study was a retrospective observational cohort study, using data from the Cardiorenal ImprovemeNt (CIN) study which was conducted in the largest cardiovascular centre in South China (Guangdong Provincial People's Hospital, China, Clinicaltrials.gov NCT04407936). A total of 88,938 patients underwent coronary angiography (CAG) from January 2007 to December 2018, and 25,027 patients were over 18 years old and diagnosed as T2DM. We excluded patients with CKD and cancer (n = 9,655), missing measurement of serum creatinine (Scr) and serum cystatin C (Scys) (n = 11,170) and follow-up information (n = 534). Eventually, 3668 patients were included (Fig. 1). The study conformed to the principles outlined in the Declaration of Helsinki and was approved by the Guangdong Provincial People’s Hospital ethics committee.

### Data collection

Data were extracted from the electronic clinical management records system of the Guangdong Provincial People’s Hospital. The baseline information mainly included demographic characteristics, medical history, medications at discharge, laboratory test results and other clinical variables. Venous blood samples were collected in the early morning after overnight fasting.

### Scr and Scys measurement and clinical definition

Scr and Scys levels were measured at admission before coronary angiography, and blood tests were conducted as routine practice by a laboratory center in Guangdong Provincial People’s Hospital.

The Scr/Scys, a surrogate marker of muscle mass, was calculated as [Scr (mg/dL)/ Scys (mg/L)]. Estimated glomerular filtration rate (eGFR) value was calculated based on Scr level using the Chronic Kidney Disease Epidemiology Collaboration (CKD-EPI) 2009 equation, and CKD was defined as eGFR < 60 mL/min/1.73 m^2^ or a medical history of CKD. Body mass index (BMI) was calculated as weight/height^2^ (kg/m^2^). Smoking was defined as smoking at least one cigarette a day for more than one year. Alcohol drinking was defined as drinking an average of two or more times per week for more than one year.

### Endpoint and follow-up

The primary endpoint was long-term all-cause mortality. All-cause death information was obtained from the Public Security and matched to the electronic Clinical Management System of the Guangdong Provincial People's Hospital records.

### Statistical analysis

Data were presented as mean ± standard deviation or median (inter-quartile range) according to the distribution of the continuous variable, and as frequencies or percentages for categorical variables. For comparisons between groups, the χ2 test or Fisher’s exact test was used for categorical variables, and an unpaired Student's t test was used for continuous variables, as appropriate.

The optimal cut-off values for Scr/Scys were determined by time-dependent receiver operating characteristic (ROC) analysis according to gender. Using the cut-off values, we separated the study population into low muscle mass (low-MM) and normal muscle mass (normal-MM) groups. The time to endpoint data were presented graphically using Kaplan–Meier (K-M) curves, and a log-rank test was used to compare differences between the groups. A multivariate Cox proportional hazards model was used to assess the relationship between muscle mass and long-term all-cause mortality. Variables that were entered into the model were carefully selected based on variables associated with known poor prognosis or variables with p-value < 0.05 in baseline or in univariable regression analysis. We also performed a subgroup analysis to assess the impact of muscle mass on long-term all-cause mortality.

Statistical analyses were performed using R, version 4.0.3 software (R Foundation for Statistical Computing, Vienna, Austria). Two-sided P values < 0.05 were considered statistically significant.

## Results

### Patient characteristics

A total of 3668 consecutive T2DM patients undergoing CAG were enrolled. Most patients were men (67.7%), and the mean age was 62.4 ± 9.9 years. Totally, 2883 (78.6%) patients were diagnosed as CAD, and 326 (8.9%) patients had congestive heart failure (CHF). There were 2206 (60.1%) patients with hypertension, 210 (5.7%) patients with stroke, 1026 (28.0%) patients with anemia, and 22 (0.6%) patients with COPD. Glycosylated hemoglobin (HbA1c) was 7.62% ± 1.60. Fasting blood glucose (FBG) was 9.28 ± 4.37 mmol/L, 2 h postprandial blood glucose (2hPBG) was 12.06 ± 4.51 mmol/L (Additional file 1: Table S1).

### Prevalence and clinical associations of low muscle mass

A median follow-up of 5 years was selected as the predict time in the time-dependent ROC analysis. The cut-off values of Scr/Scys for the best predictive value of mortality were 1.0 for men and 0.8 for women (Additional file 3: Fig. S1). According to the cut-off values, the study population were separated into low-MM and normal-MM groups. Of the 3668 participants, 2191 (59.7%) had low muscle mass. Compared with patients with normal muscle mass, patients with low muscle mass were older (63.8 ± 9.8 vs. 60.3 ± 9.7 years, p < 0.001), and had higher FBG (9.74 ± 4.61 vs. 8.99 ± 4.18 mmol/L, p < 0.001) and 2 h PBG levels (12.51 ± 4.65 vs. 11.83 ± 4.43 mmol/L, p = 0.009). Patients with low muscle mass also had lower triglyceride (TG) [1.44(1.07, 2.04) vs. 1.51(1.10, 2.16) mmol/L, p = 0.004], high density lipoprotein cholesterol (HDL-C) (0.96 ± 0.24 vs. 0.98 ± 0.25 mmol/L, p = 0.011) and low density lipoprotein cholesterol (LDL-C) (2.75±0.94 vs. 2.81 ± 0.95 mmol/L, p = 0.047) levels. The incidences of smoking, anemia and COPD in patients with low muscle mass were higher than those with normal muscle mass (39.1 vs. 35.8%, p = 0.043; 0.9 vs. 0.2%, p = 0.019; 29.7 vs. 25.4%, p = 0.005, respectively). More data on the baseline characteristics of study population are shown in Table 1.

**Table 1:** Baseline characteristics of patients in normal-MM and low-MM groups

Characteristic	Normal-MM (n = 1477)	Low-MM (n = 2191)	*p*-value
Demographic characteristics			
Age (years)	60.3 ± 9.7	63.8 ± 9.8	< 0.001
Age ≥ 60 years, n (%)	790 (53.5)	1471 (67.1)	< 0.001
Female, n (%)	489 (33.1)	696 (31.8)	0.415
BMI(kg/m^2^)	23.5 ± 3.0	24.0 ± 3.5	0.072
Smoking, n (%)	529 (35.8)	857 (39.1)	0.043
Alcohol drinking, n (%)	601 (40.7)	914 (42.7)	0.232
Medical history and clinical condition			
Hypertension, n (%)	877 (59.4)	1329 (60.7)	0.458
CHF, n (%)	129 (8.7)	197 (9.0)	0.834
CAD, n (%)	1178 (79.8)	1705 (77.8)	0.173
Stroke, n (%)	85 (5.8)	125 (5.7)	1.000
COPD, n (%)	3 (0.2)	19 (0.9)	0.019
Anemia, n (%)	375 (25.4)	651 (29.7)	0.005
Laboratory examination			
Scr/Scys	1.13 ± 0.27	0.77 ± 0.14	< 0.001
Scr(mg/dl)	0.93 ± 0.18	0.85 ± 0.19	< 0.001
Cystatin C(mg/L)	0.84 ± 0.17	1.13 ± 0.26	< 0.001
eGFR(ml/min/1.73m^2^)	83.59 ± 16.68	92.72 ± 22.89	< 0.001
FBG(mmol/L)	8.99 ± 4.18	9.74 ± 4.61	< 0.001
2hPBG(mmol/L)	11.83 ± 4.43	12.51 ± 4.65	0.009
HbA1C (%)	7.58 ± 1.57	7.69 ± 1.64	0.089
TG(mmol/L)	1.51 (1.10, 2.16)	1.44 (1.07, 2.04)	0.004
TC(mmol/L)	4.57 ± 1.26	4.49 ± 1.17	0.070
HDL-C(mmol/L)	0.98 ± 0.25	0.96 ± 0.24	0.011
LDL-C(mmol/L)	2.81 ± 0.95	2.75 ± 0.94	0.047
Medications at discharge			
OADs, n (%)	773 (53.0)	1137 (52.5)	0.788
Statins, n (%)	1313 (90.0)	1918 (88.5)	0.176
Aspirin, n (%)	1222 (83.8)	1751 (80.8)	0.026
ACEI/ARB, n (%)	618 (42.4)	975 (45.0)	0.125
CCB, n (%)	279 (19.1)	475 (21.9)	0.046

*BMI* body mass index, *CHF* congestive heart failure, *CAD* coronary artery disease, *COPD* chronic obstructive pulmonary disease, *Scr/Scys* serum creatinine-to-cystatin C ratio, *Scr* serum creatinine, *eGFR* estimated glomerular filtrationrate, *FBG* Fasting blood glucose, *2 h PBG* 2 h postprandial blood glucose, *HbA1c* glycosylated hemoglobin, *TG* triglyceride, *TC* total cholesterol, *HDL-C* high density lipoprotein cholesterol, *LDL-C* low density lipoprotein cholesterol, *OADs* oral antidiabetic drugs, *ACEI/ARB* angiotensin-converting enzyme inhibitor/angiotensin receptor blocker, *CCB* calcium channel blocker

### Low muscle mass and clinical outcomes

**Fig. 2:**
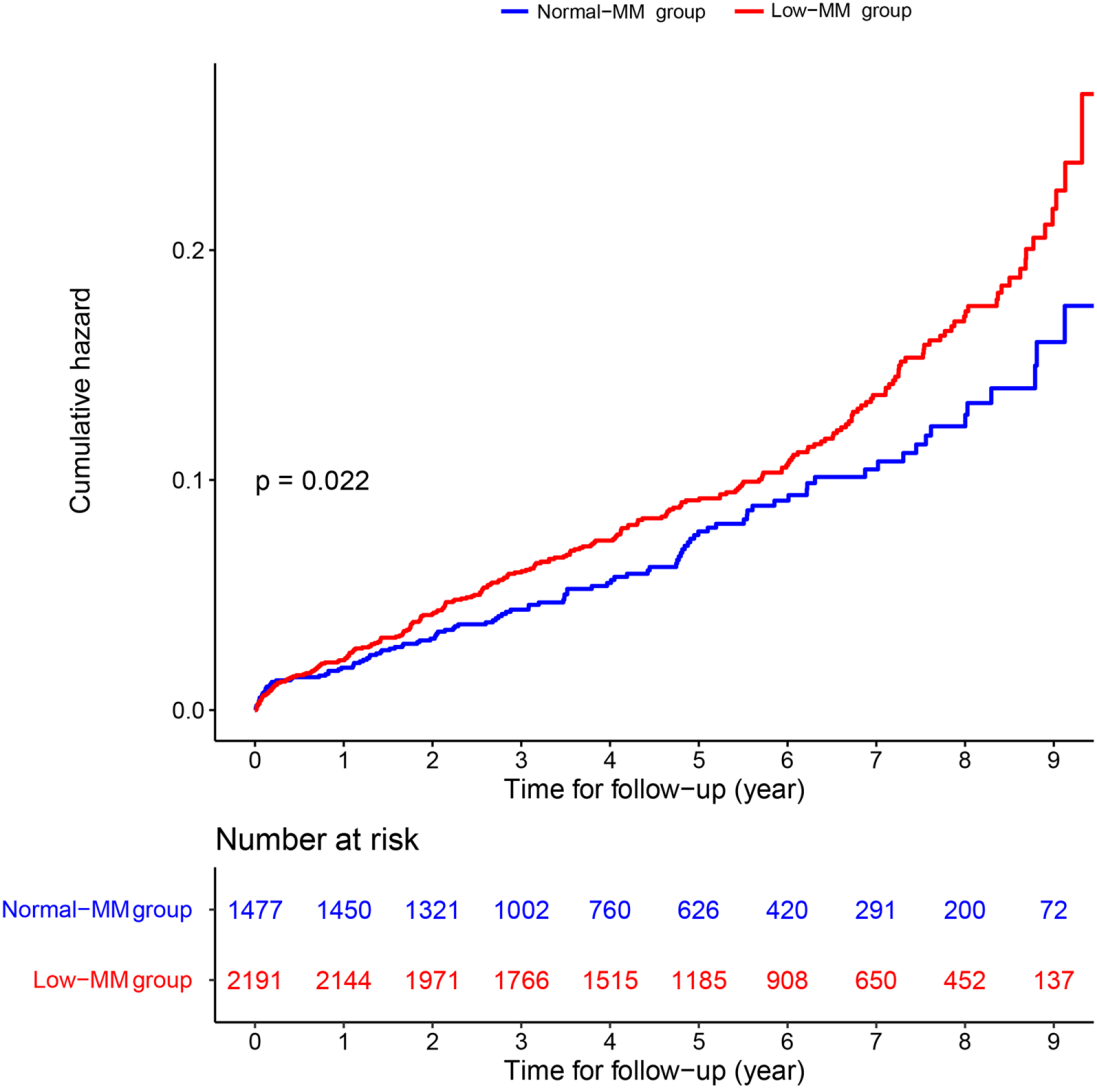
Kaplan–Meier curves of long-term all-cause mortality

During a median follow-up of 4.9 (3.0–7.1) years, a total of 352 (9.6%) patients died from all causes. The long-term all-cause mortality was higher in patients with low muscle mass as compared with patients with normal muscle mass (11.1% vs. 7.3%; p < 0.001). The time-to-event curves of long-term all-cause mortality are displayed in Fig. 2.

Multivariable analysis indicated that low muscle mass was associated with significantly increased risk for long-term all-cause mortality, regardless of whether Scr/Scys was used as a continuous variable (adjusted hazard ratio: 1.08 [95% confidence interval (CI) 1.03 to 1.13]; p = 0.009) or a categorial variable (adjusted hazard ratio: 1.36 [95% CI 1.03 to 1.75]; p = 0.021) (Table 2). Univariable analysis was shown in Additional file 2: Table S2, while subgroup analysis was shown in Additional file 4: Fig. S2.

**Table 2:** Cox proportional hazards regression analysis for long-term all-cause mortality

	Univariable		Multivariable	
	HR (95%CI)	*p*-value	aHR (95%CI)	*p*-value
Scr/Scys				
Per 0.1-point decrement (continuous)	1.05 (1.01–1.10)	0.020	1.08 (1.03–1.13)	0.009
Low-MM (Men < 1.0, Women < 0.8)	1.30 (1.04–1.64)	0.022	1.36 (1.03–1.75)	0.021
Age ≥ 60 years	1.52 (1.21–1.91)	< 0.001	1.34 (1.04–1.74)	0.024
Female	0.88 (0.70–1.11)	0.271	0.78 (0.60–1.01)	0.063
BMI	1.02 (0.98–1.06)	0.077	1.01 (0.98–1.03)	0.069
Smoking	1.18 (1.12–1.24)	0.029	1.12 (1.03–1.20)	0.048
Hypertension	1.11 (0.89–1.37)	0.355	1.12 (0.88–1.43)	0.351
CHF	1.85 (1.35–2.55)	< 0.001	1.64 (1.14–2.36)	0.008
CAD	0.80 (0.63–1.02)	0.070	0.95 (0.75–1.15)	0.091
Stroke	1.98 (1.39–2.81)	< 0.001	1.86 (1.13–2.59)	0.017
COPD	1.94 (0.73–5.21)	0.187	1.95 (0.72–5.27)	0.186
Anemia	1.29 (1.03–1.61)	0.024	1.24 (0.96–1.59)	0.097
HbA1c	1.08 (1.01–1.16)	0.034	1.09 (1.02–1.18)	0.022
TG	0.92 (0.84–1.01)	0.095	1.03 (0.92–1.14)	0.079
HDL-C	0.99 (0.65–1.52)	0.971	1.20 (0.79–1.61)	0.381
LDL-C	0.99 (0.89–1.11)	0.925	1.07 (0.95–1.21)	0.267

*BMI* body mass index, *CHF* congestive heart failure, *CAD* coronary artery disease; *COPD* chronic obstructive pulmonary disease, *HbA1c* glycosylated hemoglobin, *TG* triglyceride, *HDL-C* high density lipoprotein cholesterol, *LDL-C* low density lipoprotein cholesterol

## Discussion

In this cohort study of T2DM patients who underwent CAG, we found that low muscle mass evaluated by Scr/Scys was independently associated with long-term all-cause mortality. Patients with low muscle mass had a 36% higher risk for mortality than patients with normal muscle mass. Scr/Scys has the clinical prognostic value for long-term all-cause mortality in T2DM patients undergoing CAG.

Sarcopenia has been recognized as a disease by the World Health Organization (WHO)’s International Statistical Classification of Diseases and Related Health Problems (ICD) since 2016 [19]. The prevalence of sarcopenia defined according to the Foundation for the National Institutes of Health (FNIH) criteria was estimated to be approximately 7–20% in diabetic patients, with a variation in prevalence across healthcare settings [20–23]. The prevalence of sarcopenia using the Asian Working Group for Sarcopenia (AWGS) criteria was 15.9% in Asian diabetics [2]. The presence of low muscle mass constitutes the most critical step in the diagnosis of sarcopenia. Creatinine is an endogenous product released from muscles, and its blood concentration is dependent on muscle mass but is affected by kidney function [24]. Cystatin C is secreted by all nucleated cells, and its production and tubular secretion are uniform and not affected by muscle mass. Cystatin C is a measure of renal function and has been shown to predict glomerular filtration rate better than creatinine-based estimates [25]. Recently, Scr/Scys has been considered as a quantitative surrogate marker of muscle mass in many diseases [12–14].

There was evidence to indicate worsening of clinical outcomes when diabetes was associated with low muscle mass. A cohort study included 163 Japanese men and 141 postmenopausal women with T2DM showed that low muscle mass measured by DEXA was independently associated with all-cause mortality in female patients with T2DM [9]. On the contrary, Kruse NT et al. found that low muscle mass measured by BIA was independently associated with higher mortality in men, while neither definition of sarcopenia was associated with mortality in men or women with CKD [26]. Our large sample retrospective study, including 3668 T2DM population, showed that low muscle mass assessed by a simple indicator Scr/Scys, was independently correlated with long-term all-cause mortality in diabetic patients with cardiovascular disease or at high risk of cardiovascular disease. Our study also found that the effect of low muscle mass on outcome was statistically significant in the male subgroup and not in the female subgroup, but the interaction effect between low muscle mass and gender was not found. Therefore, further prospective studies are needed to explore this question.

The possible explanation for the association of Scr/Scys with mortality could be as follows. The muscle mass may reflect nutritional status, and definitions of malnutrition include low muscle mass within its diagnostic criteria [27–29]. Previous studies showed that malnutrition was correlated with increased mortality and cardiovascular events of acute coronary syndrome (ACS), heart failure and diabetes [30–33]. Moreover, higher cystatin C levels have been observed in patients with chronic inflammation. Therefore, lower Scr/Scys levels may be associated with inflammation [34]. Sarcopenia is known to be associated with inflammation. Inflammation is known to be predictor of increased mortality and cardiovascular events [35, 36].

As a noninvasive, easily measured and cost-effective biomarker of muscle mass and a useful prognosticator for patients with T2DM, Scr/Scys may be useful in various aspects of future clinical practice. The application of Scr/Scys to routine practice may help physicians identify patients at high risk of poor outcomes who might benefit from supplementation of protein and vitamin D and physically active lifestyles [37–39]. In addition, some hypoglycemic agents such as sulfonylureas and glinides have adverse effects on skeletal muscle, suggesting the need to circumvent the use of these drugs in diabetes patients with sarcopenia. Glucagon-like peptide 1(GLP-1) receptor agonists and dipeptidyl peptidase 4(DPP4) inhibitors seem to be favorable in protecting muscles. Although insulin can also increase muscle mass in patients with T2DM, the weight gain effect of insulin cannot be ignored, which should be carefully considered in the treatment of T2DM patients [40].

Our study has several limitations. First, our results were subject to limitations of the observational nature inherent in the retrospectively collected database. Second, we did not compare the prognostic value of Scr/Scys with DEXA, BIA, CT or MRI. However, we aimed to focus more on the prognostic value of the easily measured marker and to derive results that could be helpful in actual clinical practice. Third, since patients with missing measurement of Scr and Scys excluded, there is a possibility of selection bias. The percentage of patients with normal muscle mass was low in the present analysis can be partly explained by the bias. Fourth, we did not evaluate the relationship of Scr/Scys with inflammatory markers, and we did not investigate the changes in muscle mass over time and their association with mortality outcomes. This will require further validations in future studies. Fifth, the patients included in our study were diabetic patients with atherosclerotic cardiovascular disease (ASCVD) or at high risk of cardiovascular disease, so the results of our study could not be extended to the general diabetic patients. The prognostic value of Scr/Scys in the general diabetic patients needs to be verified in the further research. Finally, long-term all-cause mortality was complex and multivariable. Due to the lack of other endpoint events, this will limit to generalize our results.

## Conclusion

In conclusion, low muscle mass evaluated by Scr/Scys was associated with increased risk of long-term all-cause mortality in diabetic patients. The application of such a readily available indicator may help clinicians to identify diabetic patients at elevated risk for mortality and to prevent poor outcome.

## Supplementary Information

**Additional file 1: Table S1.** Baseline characteristics of all patients.

**Additional file 2: Table S2.** Univariable Cox regression analysis for variables and long-term all-cause mortality.

**Additional file 3: Figure S1.** Time-dependent ROC analysis for 5-year Mortality. A The cut-off value is 1.0 for men; B. The cut-off value is 0.8 for women.

**Additional file 4: Figure S2.** Hazard ratios for long-term all-cause mortality in different subgroups.

## Data Availability

Data relevant to this study are available from the corresponding authors upon reasonable request.

## Abbreviations

AKI: Acute kidney injury
ACEI/ARB: Angiotensin-converting enzyme inhibitor/angiotensin receptor blocker
AWGS: Asian Working Group for Sarcopenia
ACS: Acute coronary syndrome
ASCVD: Atherosclerotic cardiovascular disease
BIA: Bioelectrical impedance analysis
BMI: Body mass index
CAG: Coronary angiography
CKD: Chronic kidney disease
CHF: Congestive heart failure
CAD: Coronary artery disease
COPD: Chronic obstructive pulmonary disease
CCB: Calcium channel blocker
CI: Confidence interval
CT: Computed tomography
COPD: Chronic obstructive pulmonary disease
CIN: Cardiorenal ImprovemeNt
CKD-EPI: Chronic Kidney Disease Epidemiology Collaboration
DR: Diabetic retinopathy
DPN: Diabetic peripheral neuropathy
DEXA: Dual energy X-ray absorptiometry
DPP4: Dipeptidyl peptidase 4
eGFR: Estimated glomerular filtrationrate
FBG: Fasting blood glucose
FNIH: Foundation for the National Institutes of Health
GLP-1: Glucagon-like peptide 1
HbA1c: Glycosylated hemoglobin
HDL-C: High density lipoprotein cholesterol
ICD: International Statistical Classification of Diseases and Related Health Problems
LDL-C: Low density lipoprotein cholesterol
low-MM: Low muscle mass
MRI: Magnetic resonance imaging
NAFLD: Non-alcoholic fatty liver disease
normal-MM: Normal muscle mass
OADs: Oral antidiabetic drugs
ROC: Receiver operating characteristic
Scr/Scys: Serum creatinine-to-cystatin C ratio
Scr: Serum creatinine
Scys: Serum cystatin C
SI: Sarcopenia index
T2DM: Type 2 diabetes mellitus
TG: Triglyceride
TC: Total cholesterol
WHO: World Health Organization
2 h PBG: 2 Hours postprandial blood glucose
