# Supplementary material for: Prognostic value of creatinine-to-cystatin c ratio in patients with type 2 diabetes mellitus: a cohort study
Source: Diabetol Metab Syndr. 2022 Nov 23;14:176. doi: 10.1186/s13098-022-00958-y (PMC9686100; doi:10.1186/s13098-022-00958-y)
Supplement: Supplementary file 1 — Additional file 1: Table S1. Baseline characteristics of all patients. [file 13098_2022_958_MOESM1_ESM.docx]

**Additional file Table 1.** Baseline characteristics of all patients

| **Characteristic** | **Overall**  **(n=3668)** |  |
| --- | --- | --- |
| **Demographic characteristics** | | |
| age (years) | 62.4 ± 9.9 |  |
| age ≥ 60 years, n (%) | 2261 (61.6) |  |
| female, n (%) | 1185 (32.3) |  |
| BMI(kg/m^2^) | 23.8 ± 3.2 |  |
| smoking, n (%) | 1386 (37.8) |  |
| alcohol drinking, n (%) | 1536 (41.9) |  |
| **Medical history and Clinical condition** | | |
| hypertension, n (%) | 2206 (60.1) |  |
| CHF, n (%) | 326 (8.9) |  |
| CAD, n (%) | 2883 (78.6) |  |
| stroke, n (%) | 210 (5.7) |  |
| COPD, n (%) | 22 (0.6) |  |
| anemia, n (%) | 1026 (28.0) |  |
| **Laboratory examination** | | |
| Scr/Scys | 0.91 ± 0.27 |  |
| Scr(mg/dl) | 0.88 ± 0.19 |  |
| cystatin C(mg/L) | 1.01 ± 0.26 |  |
| eGFR(ml/min/1.73m^2^) | 89.04 ± 21.09 |  |
| FBG(mmol/L) | 9.28 ± 4.37 |  |
| 2hPBG(mmol/L) | 12.06 ± 4.51 |  |
| HbA1C (%) | 7.62 ± 1.60 |  |
| TG(mmol/L) | 1.48(1.08, 2.08) |  |
| TC(mmol/L) | 4.52 ± 1.21 |  |
| HDL-C(mmol/L) | 0.96 ± 0.25 |  |
| LDL-C(mmol/L) | 2.78 ± 0.94 |  |
| **Medications at discharge** | | |
| OADs, n (%) | 1910 (52.7) |  |
| statins, n (%) | 3231 (89.1) |  |
| aspirin, n (%) | 2973 (82.0) |  |
| ACEI/ARB, n (%) | 1593 (43.9) |  |
| CCB, n (%) | 754 (20.8) |  |

Abbreviations: BMI=body mass index; CHF=congestive heart failure; CAD=coronary artery disease; COPD=chronic obstructive pulmonary disease; Scr/Scys =serum creatinine-to-cystatin C ratio; Scr=serum creatinine; eGFR=estimated glomerular filtrationrate; FBG=Fasting blood glucose; 2h PBG=2 hours postprandial blood glucose; HbA1c=glycosylated hemoglobin; TG=triglyceride; TC=total cholesterol; HDL-C=high density lipoprotein cholesterol; LDL-C=low density lipoprotein cholesterol; OADs=oral antidiabetic drugs; ACEI/ARB= angiotensin-converting enzyme inhibitor/angiotensin receptor blocker; CCB=calcium channel blocker.
