# Supplementary material for: Prognostic value of creatinine-to-cystatin c ratio in patients with type 2 diabetes mellitus: a cohort study
Source: Diabetol Metab Syndr. 2022 Nov 23;14:176. doi: 10.1186/s13098-022-00958-y (PMC9686100; doi:10.1186/s13098-022-00958-y)
Supplement: Supplementary file 2 — Additional file 2: Table S2. Univariable Cox regression analysis for variables and long-term all-cause mortality. [file 13098_2022_958_MOESM2_ESM.docx]

**Additional file Table 2.** Univariable Cox regression analysis for variables and long-term all-cause mortality

|  | **HR (95%CI)** | ***p*-value** |
| --- | --- | --- |
| age | 1.02(1.01-1.03) | <0.001 |
| age ≥ 60 years | 1.52(1.21-1.91) | <0.001 |
| female | 0.88(0.70-1.11) | 0.271 |
| BMI | 1.02(0.98-1.06) | 0.077 |
| smoking | 1.18(1.12-1.24) | 0.029 |
| alcohol drinking | 1.02(0.81-1.29) | 0.871 |
| hypertension | 1.11(0.89-1.37) | 0.355 |
| CHF | 1.85(1.35-2.55) | <0.001 |
| CAD | 0.80(0.63-1.02) | 0.070 |
| stroke | 1.98(1.39-2.81) | <0.001 |
| COPD | 1.94(0.73-5.21) | 0.187 |
| anemia | 1.29(1.03-1.61) | 0.024 |
| FBG | 1.01(0.99-1.04) | 0.374 |
| 2hPBG | 1.02(0.99-1.06) | 0.190 |
| HbA1C | 1.08(1.01-1.16) | 0.034 |
| TG | 0.92(0.84-1.01) | 0.095 |
| TC | 0.98(0.90-1.08) | 0.728 |
| HDL-C | 0.99(0.65-1.52) | 0.971 |
| LDL-C | 0.99(0.89-1.11) | 0.925 |

Abbreviations: BMI=body mass index; CHF=congestive heart failure; CAD=coronary artery disease; COPD=chronic obstructive pulmonary disease; FBG=Fasting blood glucose; 2h PBG=2 hours postprandial blood glucose; HbA1c=glycosylated hemoglobin; TG=triglyceride; TC=total cholesterol; HDL-C=high density lipoprotein cholesterol; LDL-C=low density lipoprotein cholesterol.
