# Supplementary figures and images for: Prognostic value of creatinine-to-cystatin c ratio in patients with type 2 diabetes mellitus: a cohort study
Source: Diabetol Metab Syndr. 2022 Nov 23;14:176. doi: 10.1186/s13098-022-00958-y (PMC9686100; doi:10.1186/s13098-022-00958-y)

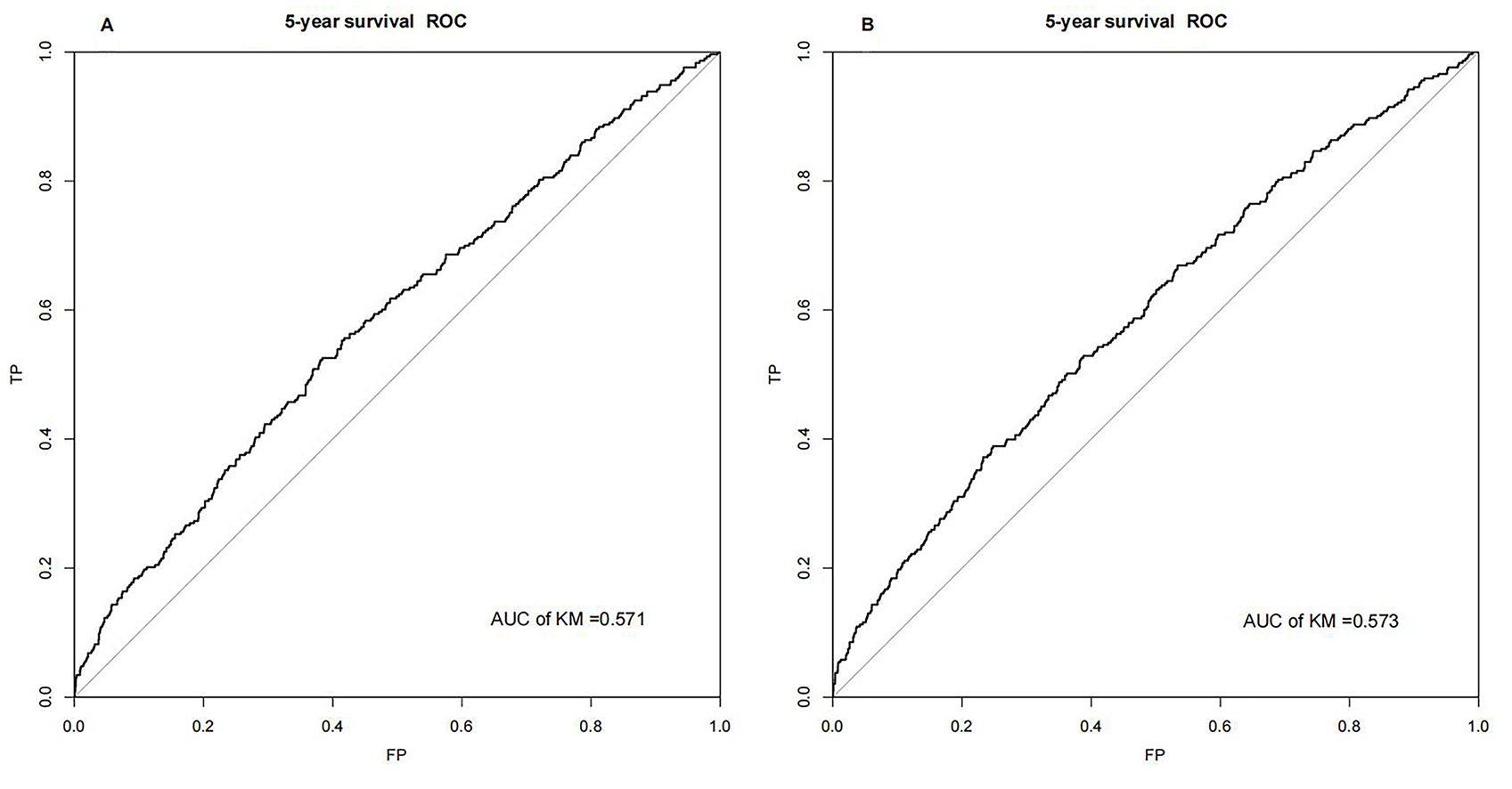

Supplement: Supplementary file 3 — Additional file 3: Figure S1. Time-dependent ROC analysis for 5-year Mortality. A The cut-off value is 1.0 for men; B. The cut-off value is 0.8 for women. [file 13098_2022_958_MOESM3_ESM.tif]

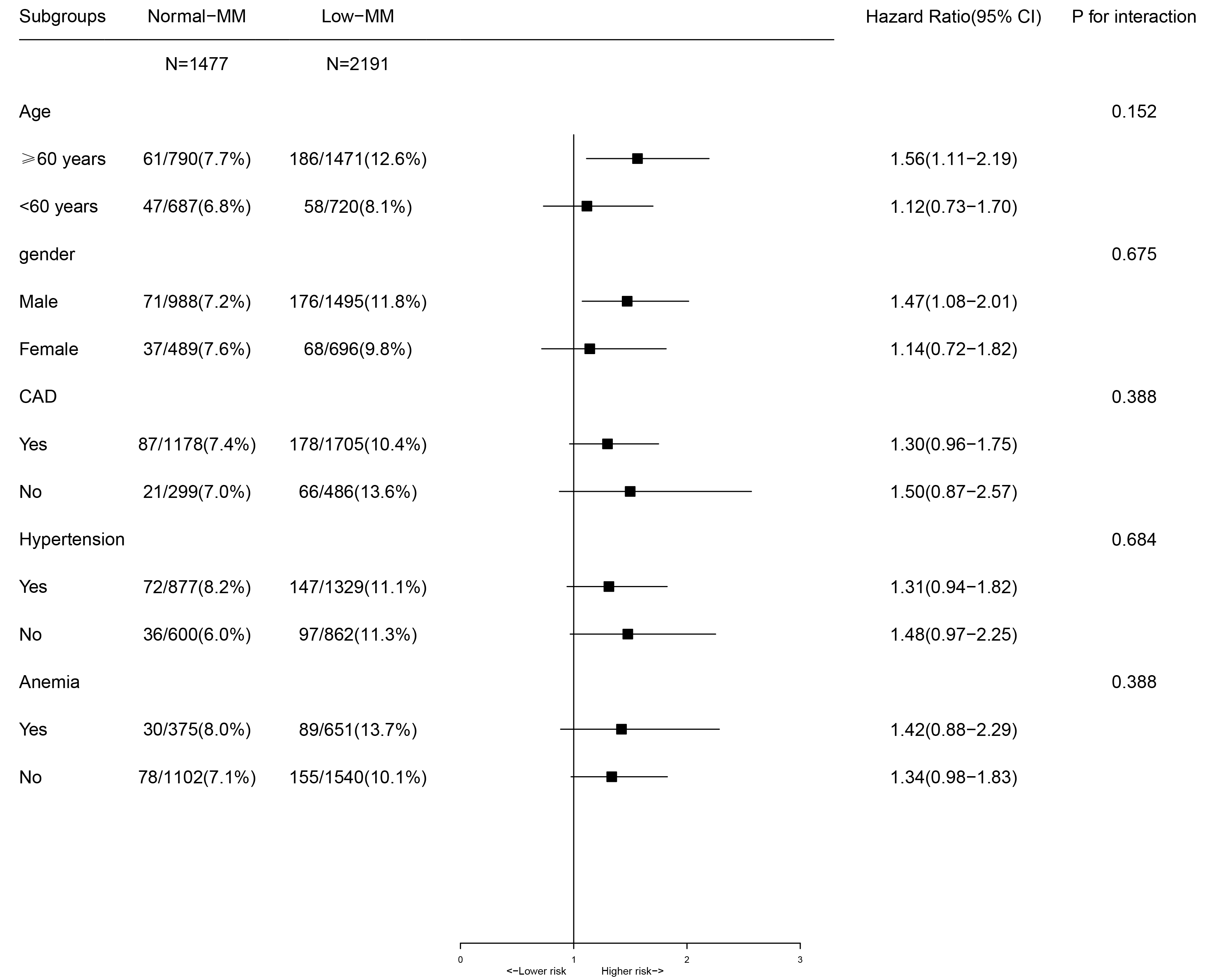

Supplement: Supplementary file 4 — Additional file 4: Figure S2. Hazard ratios for long-term all-cause mortality in different subgroups. [file 13098_2022_958_MOESM4_ESM.tif]
